# Fourth Annual Meeting of the Ohio State Dental Society

**Published:** 1870-01

**Authors:** 


### Proceedings of Societies.

### FOURTH ANNUAL MEETING OF THE OHIO STATE DENTAL SOCIETY.

### FIRST DAY.

The Fourth Annual Meeting of the Ohio State Dental Society, convened in Columbus, Tuesday, December 7, at 11 A. M., in the rooms of the Y. M. C. A. President W. P. Horton in the Chair. Prayer was offered by Rev. G. W. Phillips, of this city.

Some of the committees made verbal reports, while others were given further time to report.

On motion of Dr. J. Taft, a committee of three was appointed to present the names of candidates to fill the vacancy in the Board of Examiners occasioned by the expiration of the term of office of Dr. C. H. Harroun.

The President appointed as that committee, Dr. C. R. Taft, C. R. Butler and H. A. Smith.

During the absence of the committee, Dr. J. Taft brought before the committee the subject of the opposition made against the fee charged by the Board of Examiners to those who receive the certificate of the Board.

He said he was advised early in the session of the legislature last winter, by Mr. Brooks who was very active for two or three years in trying to secure the passage of this law, that an effort was being made for its repeal, and that unless the friends of the law were active in coming to the rescue it would probably be repealed, and that he had been informed that $25 was charged every one who received a certificate from this Board, and remarked that if that was the case he should vote for the repeal of the law himself, as he thought it was an extravagant charge, and suggested that something be done at once to meet that objection in the minds of members of the legislature.

In the correspondence between us, he said the only way in which he could reconcile the matter at all, and prevent any action being taken in the legislature was simply to promise that this society would take the matter into consideration at this meeting, and do what wisdom seemed to indicate. With that promise he would use his influence to prevent its repeal. Mr. Brooks, after considerable discussion was satisfied that it was as good as it could perhaps be made, yet he thought it would give greater satisfaction to others if the price was reduced to $5 or $10.

Dr. Decamp.—In order to bring the matter before the meeting, I move that the fee be reduced to $10. The motion was seconded by Dr. J. Taft.

Dr. Geo. Watt.—As a substitute, I move that the whole question be referred to a committee of three, to report to-morrow.

Dr. W. M. Herriott, seconded the motion of Dr. Watt. He thought no one unless it be Dr. Taft, was more fully aware of the objections made against this law than himself. He thought the law an excellent one, and believed it was doing good work for the people, and it was necessary that it be kept in existence. It was necessary for them to deliberate on this subject, and thought they were not prepared to take action now, and therefore favored its postponement until to-morrow.

Dr. J. Taft concurred in the belief that if the matter was left to a committee it might be satisfactorily adjusted.

The substitute offered by Dr. Watt was adopted and the following committee appointed by the Chair.

Drs. Watt, Jennings and Dunlap.

On motion of Dr. J. Taft, Dr. Herriott was added to the committee.

The Committee on Nominations for Examiner to fill the vacancy in the Board, reported as candidates the names of Dr. B. T. Spellman, of Youngstown, and Dr. H. A. Smith, of Cincinnati.

The President appointed Dr. N. W. Williams and Dr. I. Williams, tellers.

The members then proceeded to vote by ballot, and Dr. H. A. Smith was elected to fill the vacancy.

Dr. J. Taft announced that the treasurer, Dr. A. Berry could not be in attendance, and had sent the Treasurer's books and money in his hands by him.

On motion of Dr. Herriott, Dr. J. Taft was appointed Treasurer *pro tem.*

Letters were read from absent members regretting their inability to attend.

Adjourned until 2½ P. M.

*Afternoon Session.*

The meeting reassembled at 2½ P. M., President Horton read his Annual Address, which was well received, the speaker being applauded at the close, and a motion adopted referring his address to the Publishing Committee.

The following named persons having previously passed the required examination were elected by ballot to membership in the Society:

Drs. C. Palmer, Warren; E. C. Sloan, Ironton; H. Scott, Lancaster; M. Jefferson, Cambridge, and E. J. Way, of Sandusky.

The first topic for discussion was taken up: "Diseased condition of the soft tissues of the Mouth and their Treatment."

Dr. Blount moved that the subject be postponed until the next session.

Dr. J. Taft objected unless there was a paper to be presented on the subject.

The motion of Dr. Blount was lost.

Dr. Dunlap was called upon by the President to speak upon the subject, but declined, as he thought there were other members better prepared to do so.

Dr. J. Taft was then called upon. Said he, I don't know what I shall say in reference to this matter, but I want to know a great deal more than I do about the various affections of the soft parts of the mouth. There are many of these, and their treatment devolves upon the Dentist mainly.

The various tumors that occur in the mouth claim our serious consideration oftentimes. The condition of the mucous membrane of the mouth, the sloughing of the gums, tume-faction of the gums, and a great many points that might be named should engage our attention.

Take, for instance, the mucous membrane of the mouth, and I presume there is far more in the perversion of its functions, and the diseases to which it is subject, than any of us imagine. And I doubt not the diseases of the teeth—their decay for example, is very much influenced and modified by the condition of this tissue.

I have no doubt the mucous membrane its follicles and the entire structure of the membrane are subject to greater changes than we imagine. Its position makes it somewhat more exposed to injury than other tissues that are deeply located. It is subject to the extreme variations of temperature, and to a great variety of irritating agents, both those introduced into the mouth, and those formed there by the introduction of various foreign substances.

Now, in whatever way the mucous membrane becomes irritated or subject to this irritating influence its normal functions necessarily becomes changed, I presume when there is an incipient inflammation of this tissue that its product is changed so that it will almost invariably be injurious to the teeth, either by direct action upon them, or indirectly by a rapid vitiation of foreign substances in the mouth. facilitating the decomposition of various substances in the mouth, and the formation or production of agents that act more or less on the teeth, thus promoting decay.

I think the peculiar disturbances and affections to which the mucous membrane is subject is too much overlooked.

A little circumstance occurred a few days ago that attracted my attention to this subject. A silver plate had been put in the mouth a few days before the time of observation. It had pressed, at its posterior edge, upon the mucous membrane sufficient to redden the surface considerably. Along the line of the greatest pressure there was considerable inflammation; this extended three-fourths of an inch anterior to the line of pressure, and diminishing, so that in that space it faded out. On the silver plate was a discoloration corresponding in extent and in degree with the changed condition of the mucous membrane. The most discolored part of the plate was in contact with the most inflamed part, and as the redness decreased in the one, so did the discoloration in the other, both ceasing at the same point. Nowhere else was there the least change in the color of the plate, thus, clearly showing that the silver was acted upon, by the secretion of the irritated and inflamed mucous membrane.

The mucous glands had been so much changed as to change the secretion to such an extent as to act definitely upon the silver plate. Now this change in the secretion is of such a character as to render it injurious to the teeth either directly or indirectly.

Many of us do not hesitate to place rubber plates on the gums and allow them to be worn though irritation of the gums may take place.

I have seen cases where several of the teeth were good and it was a matter of importance to save them, and rubber plates inserted with artificial teeth, and very soon an irritation would take place so that an exceedingly irritated, reddened appearance would be presented; yet we allow our patients to go on wearing this kind of plate. In such cases it will be found that the remaining teeth will decay.

Anything that will injure or disease the mucous membrane should be discarded. This is one great objection to rubber plates, or plates that are non-conductors, as I believe the difficulty just referred to is dependent upon the non-conducting properties of the material.

Now, with regard to rose pearl. A day or two ago, I saw a mouth in which was a plate of rose pearl, in the same condition, though not to the same extent as from the rubber, exhibiting however the same redness and irritation of the membrane.

There are other affections of this membrane, among which is a thickened, corrugated or hypertrophied condition. This is usually induced by some unnatural irritating stimulus, as is exhibited in the constant use of tobacco, and similar stimulants. This condition necessarily vitiates the secretion.

There are various affections of the gums to which we ought to give close investigation, for instance, inflammation, deficiency of tone, purient growth, sponginess, a suppurating discharging condition, absorption until the support about the teeth is lost. These affections may be on the mere margin of the gums, or may extend through the mucous membrane of the mouth or extend even to the throat.

It seems to me that these are points we ought to consider, and which should in our practice claim our attention far more than it has ever done, with me at least, and perhaps with many others. I have seen cases where these conditions have existed, and been kept up, by persons wearing rubber plates. I would object to anything that would bring about any abnormal condition of any of the tissues of the mouth, whether rubber, silver, gold, platinum or anything else that would produce any such difficulties, and especially so if there are good natural teeth remaining in the mouth that would be likely to be injured in this way. I have frequently seen cases in which this affection had extended to the throat, relieved by laying aside a rubber plate. I remember now two or thre cases, where rubber plates have been removed, and gole plates introduced, when the irritation that existed soon disappeared.

Dr. Blount.—I think irritation from rubber plates depends as much on the carelessness of the patient as anything else. Sometimes the Dentist puts in a plate and the patient does not take it out for days to wash it, and the mucous membrane under it becomes diseased. I have seen cases where the surface was entirely removed.

A gentleman came to my office about a year after I made him a set of teeth on gold plate, and said he wanted to get his teeth cleaned, he had not had them out for a year, but had worn them all that time without cleaning. The condition spoken of by the Dr. was very plain in that case. We often find the gums an ashy color. In such cases I have found the treatment I have used very successful in removing it in two or three days, viz: rinsing the mouth with a mixture of chlorate of potash and honey. The proportions were, chlorate of potash, 2 drachms; honey, 1 oz.; water 3 oz. Used three or four times daily it relieves the condition spoken of as if by magic.

Dr. Spellman.—I would ask if this case spoken of had not a metallic clasp, and could not be removed?

Dr. Blount.—It was a gold clasp but could be removed very easily.

Dr. Taft.—I fully agree with Dr. Blount about the effect of impurities accumulating under the plate, it matters not what kind of a plate. But the effect arising from wearing plates that are non-conductors is a very different thing. I have seen the difficulty referred to in the mouths of persons who were the most careful and fastidious in regard to cleaning their teeth.

Dr. Keely.—Is it not your opinion that such a condition will exist under any plate when worn constantly. There is a great variety in these cases. In some there would be irritation, and some would seem simply to need rest. In some cases I have seen the most filthy plate worn perhaps for years without being removed, and the plate rubber, and yet no evil effects apparent. These cases, however, are rare. As a general rule the mouth will suffer in such cases.

Dr. Blount.—I have been very particular in making inquiries of patients who have had this condition of mouth, and have ascertained that it was generally the case with those persons who wore their plates at night. If they will take the plate out and put it in a glass of water during night, the diseased condition will rarely occur.

I wear a plate myself, and my mouth has never been irritated from it. I have worn both rubber and metallic plates; am now wearing a metallic plate.

Dr. Spellman.—It seems to me that in this matter it is only necessary to reflect that any plate that is a non-conductor of heat must result in disease to the parts covered by it, to the extent that the heat is prevented from passing off. If you confine the heat and prevent evaporation from it it will work injury, and there has been no plate yet introduced that so completely isolates the mucous membrane from the atmosphere, and all the fluids taken into the mouth as rubber plates. It is well known that this diseased condition of the mucous membrane was not observed scarcely until the use of rubber plates was so largely employed.

Dr. Rehwinkle thought the greatest trouble lies deeper than the points that have yet been touched upon. Many have by nature a constitutional condition of the mouth, an inherited condition that is commonly called scurvy. Without being produced by mechanical means it is already there, and seems to be perfectly natural in the mouths of some individuals.

The Dr. then named some instances where this inherited condition of the mouth existed, and the gums became sore, and the teeth loose.

His advice to them has been to avoid the excessive use of salts and to use more vegetable acids, take a great deal of exercise and avoid all excesses of every kind. Still this advice, though followed out, failed in one case he mentioned, and the teeth were all lost, the person now wearing artificial ones.

He related the following case, a Dentist had put in a full set of teeth on platinum for a lady in Chicago. After a while the mouth presented this difficulty, and an inflamed condition of the whole mucous membrane as far as the gum and plates came in contact. At first he thought it was perhaps because it did not fit as accurately as it might. He endeavored to remedy the difficulty by making a better fitting plate. In a short time he found it made no difference, and that it was growing worse. He then substituted gold plate for the platinum, and believed that it resulted in making a slight amendment, but not entire relief. There was no uncleanness in that case.

The speaker inquired what could be done under such circumstances, and is there no relief at all? Or, must we be content in simply giving relief? He confessed very freely to his sorrow that he had not found anything yet to give permanent relief in cases of that kind.

Then there is the "nursing sore mouth." You know what a change in the system takes place during gestation and lactation. In cases of this kind it is hard to discover the exact cause for the condition of the mouth unless you look deeper than the surface.

In the extreme sensitiveness in the gums and teeth during these periods mentioned, he had often found good results from advising simply a solution of super-carbonate of soda.

The application of chalk to the teeth during night, he had good reason to believe would check the process of decay if anything would, simply upon the theory that it will neutralize the fluids of the mouth which, during the night, have the best chance of doing the mischief.

Dr. Dunn wished to make some remarks in reference to a case which came under his observation, where plates of different kinds were worn. He had always reached these cases by the treatment he was able to bring to them. If a healthy mouth were affected by the material which covers the mucous membrane, we should seek for that material which will best control or remedy the difficulty. A case had come under his observation in Columbus, where a lady had worn a gold plate, and though in a measure satisfactory, it was, however, exchanged for a rubber. This was a full denture, upper and lower, and the rubber plate could not be worn with satisfaction during the day and night. If worn at night the mucous membrane appeared so much diseased, that it was necessary to lay the plate aside during the day. The exchange for the mineral plate has nearly obviated the difficulty, so that the patient wears it night and day ; and the diseased appearance of the mouth is gone. It seems to me, our best results are reached, by securing something that can be worn by night and day, for artificial dentures, as best meeting the wants of our patients.

Dr. Blount asks if any have had experience in treating a condition of the mouth where there is a complete absorption of the alveolar process, where the inflamed condition of the gums is such that they are of a dark purple ; and the process being finally, entirely absorbed, the teeth drop out, though perfectly sound. This condition, which he had noticed, was hereditary, and you will see it in the father, mother and children. He would like to know the method of treating cases of this kind.

Dr. Watt thought in hereditary cases, where the absorption of the alveolar process was accompanied by a sponginess, in a very large majority of the cases, the patient is gone beyond remedy. We often see young persons thus afflicted, where there has been neglect ; where they have, perhaps, been confined in a close school room, and in a bad atmosphere, but still, there may be in them vitality enough to afford hope for relief. He had seen a young lawyer, who had inherited a good many things, consumption, scrofula and other diseases, and finally died with a typhoid fever, before either of these other diseases had time to kill him.

He said, when he commenced treating that young man, he had twenty-eight teeth in his mouth, all sound; yet he had been advised to have them all extracted, and artificial ones put in. After treating him some time, the teeth all became tight again; the upper teeth were denuded, yet they were made quite solid by the treatment. It became necessary to take the patient out of the school room into the open air, to put him in proper condition. We do not want more brain or nerve in such patients, but fibrous tissue; and he knew of no better food for the patient than beef steak, as it contained the very element needed. Eggs are not good, as there is too much albumen in them. He recommended a solution of chlorate of potash as a good wash; and said, another thing that acted well was simply white sugar. One good quality of sugar is its cleansing property. That is the reason it is an ingredient in the popular salves. It is a solvent, and dissolves away the matter, and it is for this reason a good mouth wash. If we had to have but one thing to wash the mouth, sugar would be that one best thing.

The gums, in the cases referred to, must be watched, and the vessels depleted if necessary. This will especially be the case if this dark color appears. Some use a lancet for this purpose, but a little flat excavator, made extremely sharp, is best with which to prick the vessels of the gums wherever this full appearance is seen--not every one, but a great many. As a general rule, the treatment is too active, as in such cases there is very little vitality.

The cases refered to must be taken in time. We have many times, in beginning treatment, told parents that their children must be taken out of school, in order to secure the full benefit of the treatment. In one case in Xenia, he told a man that he must take his boy out of school. He was unwilling to do so, and the doctor told him if he did not, he would report him to the authorities of the church to which he belonged. The boy was not as large as his school-mates, four years younger. He was about thirteen years of age, and was in classes with boys of nineteen and twenty, in the High school. The parents, seeing that he was in earnest, took their boy out of school. He got out into the open air, frequently riding on the top of his father's omnibus, and making himself miscellaneously useful about his father's livery stable; and he grew up a well developed young man.

At the time the treatment commenced, one of his teeth, a first molar, was decayed and troubled him some. On examination, it was found to be loose. By taking hold of it, and shaking it, the second molar would shake also. The bicuspids were also loose, and the mouth generally in a very bad condition.

In such cases the great aim should be to build up the fibrous tissue, by prescribing the proper nourishment, exercise, etc.

Question—What is the condition of the teeth in that man's mouth to-day?

Dr. Watt—The second bicuspids were good a short time ago, as were also the posterior molars. Before treatment commenced, almost the whole mouth was rotten, and the breath smelt like sulphuretted hydrogen.

Dr. C. R. Butler said, I am reminded every time we come together, in this Association, that we lack knowledge. We can go about so far, then we begin to ask questions.

I do not presume to be able to say what shall be done in many of these cases that have been presented.

Considerable has been said in reference to the diseased conditions of the mucous surface of the mouth, arising from local causes, as to how they may be overcome, etc. One word in reference to the use of rubber plates for artificial teeth. It has done more mischief than any other substance that has been tried in the mouth, and it would seem that simply its abandonment was the remedy. The most difficult cases we have anything to do with, are where inflammation and irritation arise from general and constitutional causes, and it is folly to attempt to reach them by simply local treatment. We may palliate them, it is true, but, so far as a cure is concerned, it never can be done by local treatment. I would just refer to one case, as it is necessary, sometimes that we should have a sort of test.

Dr. Watt has said that many of those cases of diseased condition of the mouth are from close confinement in the school room; and that an entire change in the whole *regime* must be instituted before we can have any beneficial results from our efforts in treating them. We have seen this tendency to a spongy condition of the gum, causing great tenderness of the teeth, so that beating upon them, or brushing them was attended by pain; and that with persons who were very particular in cleansing their teeth, and caring for them; and from close observation I find in a majority of these cases there is a general depression of the vital powers of the whole system. Such persons should change their mode of living; and especially should they take plenty of out door exercise; and then we may find a very marked change, with a very slight local treatment.

He mentioned a case that had come under his care at Cleveland, and afterwards under the care of Dr. Dixon, of New York, and afterwards came back. The patient had been advised to take good, nutritious diet, and plenty of out-door exercise; and not to confine himself too much to his business; and simply to use alkali washes in the mouth. There is an acidity, peculiar to the secretions of the mouth, that seems to favor irritation, and keep it up.

He was glad to hear Dr. Watt say it was necessary to be very careful how we deplete the gums. To just take a knife and slice all the way around is barbarous, and will do more harm than good.

Dr. Watt had said sugar was good for the teeth. Why? Because it is a solvent. It is something more. There must be something to solve before it is a solvent. It excites a flow of saliva, and causes impurities to be washed away. Anything you could put in the mouth that would thin down the fluids, would act the same as sugar; but sugar being so pleasant to the taste is, perhaps, better.

Some claim that the alveolus can be restored entire where it is gone; but he had not much faith in this. There are many things recommended as remedial agents. One thing he had found very salutary, viz: precipitated chalk with a little orris root, about one ounce of the latter to two of the former, made into a paste, and brushed in and left on the teeth when going to bed. It seems to neutralize, or keep under the secretions of the mouth at night, better than anything he had ever tried.

Dr. Dunlap thought attention enough was not given to the prevention of tartar upon the teeth. Dentists do not instruct their patients with regard to the formation of tartar on the teeth as they should.

There is also the use of tobacco which is very deleterious in its effects upon the membranes of the mouth. Smoking is also injurious. He had seen cases where this habit had caused the mucous membrane to be entirely drawn up, presenting a starved appearance. But he thought the greatest omission in the discussion of the subject was the non-attention to the removing of tartar from the teeth.

*Evening Session.*

The Society met again at 7½ P. M. President Horton in the Chair.

On motion of Dr. G. W. Dunn, the reading of the minutes was dispensed with.

The discussion of the first topic was continued.

Dr. Horton, having called a member to the Chair, spoke upon the question. He said he confessed he had in many instances been baffled in the treatment of diseased conditions of the soft tissues of the mouth, more particularly from a difficulty in getting patients to do what he prescribed, than from a want of knowledge on his part as to what caused the disease. In several instances he had been obliged to call in some of his medical friends in whom the patients he was treating had great confidence, before he could get them to admit that there was any trouble back of what was shown on the mere surface.

Dr. Watt commends the right method. And in order that we may know what is required in these cases we need to devote more study to these things. We ought to consider ourselves a branch of the medical profession. If we do not understand the nature of these cases, in forty-nine out of fifty, we will be entirely at a loss what to do, and the patient will not be benefitted by us.

In one instance a lady came seven or eight years ago to him for advice, but she did not think best to take his advice. He called her physician and held a consultation with him, but she would not heed their advice, but went and had her teeth extracted. The difficulty became complicated and a year or so ago she died from the effects of it, though the difficulty might have been remedied had the patient pursued the course advised. The difficulty was back of the teeth.

He thought a diseased condition of the mouth resulted sometimes from atmospheric causes. There are times when there seems to be an epidemic tooth ache, when it seems that almost every one who has decayed teeth will complain of what they call tooth ache. This difficulty arises from atmospheric causes, but what they are in particular he was not yet certain.

He thought another cause was a torpid liver, and where the digestive organs become impaired. The person should take proper exercise, and his habits of life should be such that the food will be properly digested and conveyed to the spot needed.

Some of these things it may be impossible for us to regulate in our patients immediately. They claim that the trouble is only local, and demands only local treatment. He had several times within the last few years, and especially during the past fall, recommended his patients to their family physician to enquire into this matter.

In the medical, as in the Dental profession, men are afraid to pursue a specialty. You find the gentleman who undertakes it dubbed a quack by the very persons who ought to encourage him. He knew men who had spent thousands of dollars in purchasing microscopes and other instruments to examine the secretions, etc., so as to understand more perfectly such things, and the first thing, perhaps, they would be dubbed quacks by the gentlemen of the profession. We should pursue a steady course in investigating any special subject, no matter who may object or cry out against it.

This matter of salivary calculus, spoken of before, is a great source of irritation at some times. It may be caused by local irritation in the first place. This requires immediate removal.

The treatment of patients should be gentle, and they should not be dismissed with a single examination, but should be seen again, so that the irritation may be removed to our satisfaction, and that we may find out what other treatment may be needed.

He mentioned the following case:

A lady had written him in relation to getting a set of artificial teeth. She had been using rubber plate which produced such irritation that she could not wear it, and had employed some one to make her a porcelain plate, but that did not satisfy her, and she desired to know my terms for making her a gold plate.

She lived some distance from the city. After some correspondence she came to his office, and the moment he saw her, said he, "I set my eyes upon a hypocondriac." She was a woman who had tried all the medicines ever invented. I think the loss of the teeth was the result of highly inflamed gums produced by mercury. Her physician advised the extraction of the teeth.

Mr. Bacon himself—the Goodyear Bacon, the Dental Vulcanite Bacon—said that he could not wear rubber having been salivated previously. This lady had been salivated by the mercurial preparation, and could not wear them. She also had chronic catarrh, which not only affected the mucous membrane of the mouth, but extended to the throat.

I made her gold plates, and in about a month her husband wrote me that it was a perfect success, which was much better than I ever expected. But the lady instead of entirely recovering her health, died in a few months. She was diseased from head to foot. What I learned of her suffering and final demise, proved to me what I thought at first, that the case ought to have been treated years before intelligently.

Cases are constantly occurring that need general treatment, and the only way we can do is to inform ourselves what the cause of the difficulty is and how to treat it. If we can not find intelligent physicians to go to work, let us take it up ourselves without fear of being called quacks or dubbed empirics.

Sometimes, if you send a case to a physician for treatment he will make light of it, and tell the patient some quack Dentist sent them.

The difficulties that arise from the use of rubber plates are more complicated than we are apt to imagine or look for. He had known one or two instances where the mouth of the person had been injured by the pressure or heat, so that he was obliged to do all the masticating on six or eight teeth, with the help of the artificial teeth, until the other teeth affected by the pressure of the plate had been entirely destroyed.

He accounted for this trouble in two ways. One was from the mere pressure on the gums, and the other, that there was a holding in of the heat, until it produced entire absorption.

With silver or gold plate he had seen nothing like the same difficulty in this direction, as attends the use of rubber plates.

Dr. Watt said he intended to speak of fibrous tumors, when up before. These sometimes grow to such a size that the person can not shut his mouth. Such a case was described in the Mississippi Valley Association.

Dr. Watt said he had seen, and removed quite a number. They can be cut off without hurting the patient much. It is generally, he thought, a case of genuine hypertrophy. He never had any difficulty in stopping hemorrhage. What he rose for was to advise all those that found the family physician such a terrible person to remove to Xenia. There they are in the habit of referring cases of diseased mouth to the Dentist. He did not know but that Dr. Taft trained them when he was there, while they were young.

Dr. Buffett said he must stand up for the physicians of Cleveland. In treating cases of neuralgia they sent for the Dentist and counseled with him, before advising what to do. He thought they were favored in regard to physicians there.

Dr. Horton said he would bear Dr. Buffett out in saying what he did, in regard to a few physicians there, but he was not acquainted with all the physicians in town. There are many scattered over the city that will not do as those to whom he referred.

A few of their physicians had become very liberal in this respect. He knew of them having been called upon to treat neuralgia—facial neuralgia and sympathetic neuralgia—and having failed in their treatment, and have learned by sad experience that knowledge was not going to die with them. They are approachable, but it is a difficult matter to reach all the physicians.

He had had occasion to remove one or two fibrous tumors in the past few weeks. One in particular he remembered, where it reached from the superior right bicuspid to the canine tooth, growing between these teeth, and down until it almost covered the crowns of the teeth. He found no difficulty in taking a small knife and cutting it off smoothly. He stopped hemorrhage in, perhaps, a rather barbarous way.

He cauterized it, having made an instrument before he took the tumor off, that just about covered the place, and heated it, and as soon as he cut off the tumor stopped the blood by putting it right on the spot. It was thoroughly eradicated, and is very nearly healed. He had tried several in that way, and found the cautery the best thing to put on.

Dr. I. Williams, said he would like to have some information in regard to some cases that had come under his observation.

One case was that of a lady who was wearing a plate, and on the alveolar ridge there appeared, perhaps a year ago, a tumor resembling a wart. It continues to grow, and as it grows it flattens out until the surface has become, perhaps, as large as a dime, with a very narrow base, and still seems to be growing rapidly.

The other case, he said, was like this: A physician called on me a few days ago, stating that he had a patient whose left tonsil was entirely petrified, and wished to know if I had any instrument with which it could be removed. I told him, I thought I had, and he said he would bring the patient up the next day. On the next day the patient came in. I discovered on the tonsil a rather whitish spot and touched it with an instrument, and found it to be a substance resembling salivary calculus, very hard and about the size of a common hickory nut imbedded completely in the tonsil. The patient is almost unable to talk but is fearful about having an operation performed.

Dr. Butler inquired what he considered to be the nature of the trouble.

Dr. Williams replied, that he considered it to be of the nature of lime. He thought it resembled in character the accumulations of lime found in the bladder sometimes. The patient had suffered from diptheria. A physician with whom he talked in reference to the case, said there was a deep cavity left when the patient recovered from diptheria.

Question by a member—Did you make an effort to punch it, and did it seem fast at the base?

Dr. Williams.—I think it is simply imbedded in the substances of the tonsil, and believe it could be easily removed if the patient would allow it.

Dr. Watt remarked that such calculi are very common and are composed mainly of carbonate and subphosphate of lime.

He had, eleven years ago, seen a tumor weighing three ounces and a quarter, and had broken off a piece and analyzed it. He thought the tumor referred to could be easily removed, and that it should be done.

Dr. Williams.—I would like to have the opinion of some one or more with regard to the first case I mentioned.

Dr. Butler.—Where is it located?

Dr. Williams.—About the point of the second bicuspid.

Dr. Butler.—If it was my case I would cut it off.

Dr. Rehwinkle.—I would carefully cut around the base of the tumor down to the bone, and think that would do away with the tumor.

Dr. Butler said he would like to refer to a case that came under his observation some two months ago. The case was that of a lady wearing an entire denture. A tumor had grown out a little to the right of the mesial line, and become so painful that she was unable to wear the plate, but had persisted in doing so, until it became intolerable. He had known her for some seven or eight years and she had worn a plate during that time. He examined the tumor and told her there was something there that would have to be removed. She got her fears excited and thought it was some cancerous difficulty.

He advised her to have it attended to immediately. It passed on two or three weeks, when it began to discharge considerable matter. He examined it again, and by passing an instrument into it found a hard substance. She agreed to have it removed, and he administered chloroform the next day and cut down to the hard substance and took it out entire.

Dr. Geo. W. Dunn said this reminded him of a case where a wisdom tooth had been imbedded in the gum. He understood that an effort had been made to extract the tooth before the plate was inserted. The bearing of the plate had irritated the tooth and it became thoroughly imbedded in the gum. It had been pronounced by a physician an irritation of the bone. The speaker said in examining it, and as in the case Dr. Butler referred to, he found a hard substance and took out nearly a full sized wisdom tooth, which had been almost entirely out of sight for years.

Dr. J. Taft said the subject, in some of its aspects, had been pretty thoroughly discussed, especially as far as isolated cases were concerned. Such cases are always interesting, but there is a foundation principle underlying these that is of much more value. He would like to refer to two or three other points if the time were not so far spent. There is a loss of the gums; and there is tumefaction of the mouth in various places; then the nursing sore mouth, which has been referred to, but no certain principle underlying it has been discussed.

He would like to hear all those discussed thoroughly, and go down to the principle on which they are based, then we have the foundation and may understand very correctly all these varieties.

Without these foundation principles we may recite cases from now until doomsday, and know nothing about them any further than the recitation of the cases goes.

The question of nursing sore mouth is a very interesting one, because it is many times exceedingly annoying to the mother and children, the nature of which he acknowledged he did not thoroughly understand, but he believed it occurs because of a reduced condition of the vital forces of the system. Sometimes a slight irritation in the mucous membrane will cause a difficulty when there is feeble vital force. Imperfect nutrition may also cause it. These two things are, perhaps, the principal causes. There is also a letting down of the vital forces from the nourishment imparted from the mother to the child during lactation.

Another very interesting point in regard to this last affection is the transmission of it from the mother.

He was glad to hear two or three gentlemen refer to tumors. One fortunate thing is, that they are not of very frequent occurrence, especially in an injurious or malignant form He would have been glad to have heard that point more fully discussed. Cases are often brought to the Dentist for his opinion as to their character, etc. You may be asked, do you think this a cancer? Will it grow rapidly, or will it grow much worse? What will be the final result of it? We too often have to say "I don't know," to all such questions. He did not propose to discuss these questions now, but simply referred to them.

Something had been said in reference to constitutional causes in their effects upon the teeth and mouth. There may be a predisposition in the system favoring any diseased condition here as with other parts of the body. This original trouble may have occurred with the child itself when a fœtus in the uterus, or at its birth, or it may have been transmitted from the parent to the child.

In reference to the manner in which Dentists may be approached by physicians, he thought in many cases what Dr. Horton had remarked proved true, while in other cases the Dentist receives more consideration than he deserves from the physician. If the Dentist has ability he will soon be known, and the better class of physicians will accord to him all he deserves. If, for instance, when the Dentist is called to consult with a physician, and shows palpable ignorance on his part in reference to the case, how can the intelligent physician accord to him credit for professional learning. And how much more should be known by many of us in regard to many things belonging to the profession that we do not know. We are exceedingly ignorant about many things which we should understand, and we betray that ignorance when we come to talk with physicians upon those subjects. If we simply comprehend how much ignorance we have we would not blame any one for not giving us more credit. If we would delve down deep for the truth; investigate the matter of histology and physiology—and here, by the way, we must pay a compliment on some men in the Dental profession for the investigations they have made in that direction; there are not many of them, but there are some, and if a majority of us would do that, the medical profession would accord to us more than we would ask.

Dr. H. A. Smith said frequent allusion had been made during the discussion to the reddened or inflamed condition of the mouth, resulting from wearing artificial plates. He had seen this condition, and believed wherever he observed it in patients wearing metal plates, it had been occasioned by neglect. He had in many instances observed it where rubber plates were worn, sometimes the result of uncleanness, but he had known it in other cases where the patients were scrupulous about cleansing their plates.

It occurred to him it might arise from the coloring matter of the plates, though the coloring matter was considered entirely inert. The American Medical Association had appointed a committee to report on this subject, but the report was never made.

A case presented itself to him about six months ago where there was a violent affection of the mucous membrane. There was a fullness of the vessels of the gums, and a red ridged condition marked through the whole surface covered by the rubber plate. The patient was advised at once to change the plates, and he advised rose pearl to be used as he was about that time experimenting in that direction. He made rose pearl plates for the patient, and in about two months there was a decided improvement. Perhaps it was because those plates were non-conductors that they caused the trouble. He knew it was thought that the mercury in the rubber plates might induce this condition, but of that we are not certain. He was quite sure, however, that with plates that were not non-conductors we have better results.

Dr. G. W. Keely remarked that it had been his experience with regard to the effect of the plates on the soft tissues of the mouth, that at least in all ordinary cases where he had seen the condition of mouth referred to it was where the patient wore the plates constantly, only taking them out to clean them after eating.

He had known cases to occur where the patient had kept the mouth perfectly clean, but who gave the mouth no rest. He thought all would agree that when the plates were thus worn constantly, and the mouth having no rest, such a condition would be likely to be the result.

Dr. Rehwinkle, desired to add a few words from personal experience. He had been wearing silver plates, gold plates, and for a short time porcelain plates. He thought there was everything in wearing plates continually, or leaving them out at night. He had no doubt his mouth was in a healthy condition, but if he wore his plates continually, he would soon have inflamed mouth, but by leaving them out at night he never found any difficulty.

So far as comfort is concerned he must give preference to rubber plates, even to gold, but it must not be understood, that he would recommend rubber over gold in all cases. He spoke only of his own case.

More breakages of plates occurred at night than at any other time. A great many persons are apt, when they go to sleep, to see their grand mothers, and when they do, they are very apt to grind their teeth (laughter).

Dr. Maxwell desired to give an account of a special case that came under his observation a little over a year ago. A lady who had been wearing a rubber plate for some time, and before that a gold plate with a full upper denture, had experienced this annoyance during the wearing of both plates. She wore her plates continuously. Said she could not rest well at night without them. It had been his practice to let patients consult their own convenience in this respect.

He recommended to this lady a porcelain plate. She has been wearing it about thirteen or fourteen months, and had written him that she wears it continuously except when taking it out to clean it, which she does very frequently. From an examination of that mouth since, he found it entirely free from that inflamed condition.

The opinion he formed from that case was that porcelain plate is free from whatever it is that produces that effect which he believed belonged more particularly to rubber plate.

He had one or two other cases which he had observed closely, where the effect was not so marked, though there was a decided improvement where the porcelain had been substituted for rubber.

On motion of Dr. Watt, the discussion on this subject was closed.

The Secretary read a communication from a delegate—J. G. Templeton, Pennsylvania—who was appointed by the Pennsylvania Dental Society to attend the meeting of this Society.

On motion of Dr. Rehwinkle, his credentials were ordered to be placed on file.

[To be continued.]

---

Order of Business for the twenty-sixth annual meeting of the Mississippi Valley Dental Society, to be held in Cincinnati, commencing Wednesday, March 2d, 1870:

1. Organization at 10 o'clock A. M.

2. Calling roll, and reading extracts from minutes of last annual meeting.

3. Reports of officers.

4. Election of officers at 3 o'clock P. M., Thursday, March 3d.

5. Essays and papers to be read at the opening of discussions of the respective subjects.

6. Subjects for discussion:

Relations existing between Patient and Operator;

Dental Quackery and its Remedy;

Causes of Dental Caries;

Heavy Foil and Heavy Mallets;

Disease of the Gums and Alveolus, and Treatment;

Miscellaneous. Exhibition of Instruments and Appliances for one hour, commencing at 1½ o'clock P. M.—second day.

